# Artificial Intelligence-Powered Chronic Obstructive Pulmonary Disease Detection Techniques—A Review

**DOI:** 10.3390/diagnostics15202562

**Published:** 2025-10-11

**Authors:** Abdul Rahaman Wahab Sait, Mujeeb Ahmed Shaikh

**Affiliations:** 1 Department of Archives and Communication, Center of Documentation and Administrative Communication, King Faisal University, P.O. Box 400, Hofuf 31982, Saudi Arabia; 2 King Salman Center for Disability Research, Riyadh 11614, Saudi Arabia; 3 Department of Basic Medical Science, College of Medicine, AlMaarefa University, Diriyah 13713, Saudi Arabia; smujeeb@um.edu.sa

**Keywords:** chronic obstructive pulmonary disease, artificial intelligence, lung disease, medical imaging, computed tomography, chest radiography, spirogram, computed tomography

## Abstract

Chronic obstructive pulmonary disease (COPD) is a progressive respiratory condition, contributing significantly to global morbidity and mortality. Traditional diagnostic tools are effective in diagnosing COPD. However, these tools demand specialized equipment and expertise. Advances in artificial intelligence (AI) provide a platform for enhancing COPD diagnosis by leveraging diverse data modalities. The existing reviews primarily focus on single modalities and lack information on interpretability and explainability. Thus, this review intends to synthesize the AI-powered frameworks for COPD identification, focusing on data modalities, methodological innovation, evaluation strategies, and reporting limitations and potential biases. By adhering to Preferred Reporting Items for Systematic Reviews and Meta-Analyses (PRISMA) guidelines, a systematic search was conducted across multiple repositories. From an initial pool of 1978 records, 22 studies were included in this review. The included studies demonstrated exceptional performance in specific settings. Most studies were retrospective and limited in diversity, lacking generalizability and external or prospective validation. This review presents a roadmap for advancing AI-assisted COPD detection. By highlighting the strengths and limitations of existing studies, it supports the development of future research. Future studies can utilize the findings to build models using prospective, multicenter, and multi-ethnic validations, ensuring generalizability and fairness.

## 1. Introduction

Chronic obstructive pulmonary disease (COPD) poses significant risks to public health across the globe, ranking among the leading causes of morbidity and mortality [1,2,3]. Due to the complex nature of early symptoms and the underutilization of spirometry, the condition is frequently unrecognized [4,5]. The widespread adoption of spirometry is limited by several factors, including the limited availability of equipment, the need for competent technicians, and patient effort constraints, which can result in delayed diagnosis and loss of opportunities for timely intervention [6,7,8]. Although imaging modalities, including chest radiography (CXR) and computed tomography (CT), symptom questionnaires, and electronic health records (EHRs) are used extensively in clinical workflows, these modalities are insufficient to serve as standalone diagnostic tools for COPD diagnosis [9,10,11]. Early diagnosis of COPD is crucial for improving patient outcomes. However, it remains a persistent challenge in routine practice due to the shortcomings of traditional diagnostic approaches [12,13,14,15].

Recent advances in artificial intelligence (AI) offer novel prospects for COPD screening [16,17,18]. By using massive amounts of complicated clinical data, AI can identify subtle disease signatures [19]. At the imaging level, deep learning algorithms can identify anatomical abnormalities associated with COPD, such as emphysema and airway remodeling [20]. Analyzing respiratory sound recordings with convolutional neural networks (CNNs) enables the automatic classification of airflow obstruction [21,22,23]. EHR data, including structured factors (demographics, smoking history, comorbidities, and laboratory tests) and unstructured clinical narratives evaluated through natural language processing, can be utilized for phenotyping and risk prediction [24]. Recently, wearable and physiological signals, including capnography, photoplethysmography, and nocturnal oximetry, have been investigated as potential low-cost alternatives for COPD identification [25]. The wide range of modalities highlights the significance of AI systems in enhancing conventional diagnostic processes [26,27,28]. The performance of the AI applications relies on the quality and type of features extracted from these diverse modalities [29]. AI’s clinical decision support capability makes it crucial for identifying COPD [30]. The integration of probability scores, predicted lung function indicators, and customized risk profiles enables AI models to generate meaningful outcomes, supporting clinicians in making effective decisions [31,32,33,34]. It enhances triage decisions, accelerates treatment, and reduces underdiagnoses in primary care [35]. AI models can benefit from multimodal fusion, analyzing imaging, physiological, acoustic, and electronic health record data to better understand disease heterogeneity. In addition, AI applications have the potential to correlate imaging-derived features to exacerbation risk and mortality, highlighting their relevance in long-term therapy [35]. These advancements emphasize AI as a key breakthrough for redesigning COPD screening, staging, and monitoring.

In the context of AI-based COPD screening, several review studies have been conducted. However, these studies have significant shortcomings, which limit their applicability in guiding research and clinical translation. The majority of the reviews have a limited modality focus, focusing solely on imaging-based approaches (CT or CXR) or limiting their application in AI-powered COPD screening approaches [36,37,38,39,40]. Furthermore, these studies are unable to incorporate diverse data sources, such as respiratory sounds, electronic health records (EHR), and wearable physiological signals [41,42]. The existing studies emphasize algorithmic performance indicators rather than rigorously evaluating the model’s effectiveness. The need for external validation on separate cohorts to determine a study’s practical usefulness is overlooked in existing reviews. Furthermore, implementation, fairness, and explainability are underemphasized in the existing studies. A limited number of studies explore algorithmic bias, subgroup performance differences, or deployment equity across demographic or geographic groups. Likewise, the integration of workflows, cost-effectiveness, and real-world viability is underexplored. The existing reviews lack in reporting methodological standards, knowledge gaps, or significant advancements, such as multimodal fusion, semi-supervised learning, and uncertainty quantification. These factors motivate us to conduct this review.

This study intends to investigate the potential of AI applications for COPD identification using imaging, spirometry, respiratory sounds, EHR, and physiological inputs. Unlike existing studies, it assesses methodological rigor, validation quality, and deployment readiness, bridging the gap between technological advancement and clinical application. It provides researchers, clinicians, and policymakers with a consolidated evidence base, presenting a set of actionable recommendations for advancing AI in the identification of COPD.

The remaining part of this study is organized as follows: Section 2 presents the review protocol, including the databases, searching strategy, and eligibility criteria. It explains the screening process based on the PRISMA guidelines, including the extraction of study characteristics, AI approaches, datasets, validation strategies, outcomes, and the assessment of risk of bias. Section 3 highlights the findings of this review. It discusses the role of methodological innovations, dataset diversities, and generalization, contextualizing the study findings with current clinical practice and research gaps. Finally, Section 4 provides key insights, underscoring the potential of AI in identifying COPD. It outlines the study’s contribution and provides recommendations for future research.

## 2. Materials and Methods

The purpose of this study is to synthesize existing research on the use of AI for detecting COPD. In accordance with the updated Preferred Reporting Items for Systematic Reviews and Meta-Analyses (PRISMA) guidelines [43,44,45], we extracted and reviewed the studies associated with AI-driven COPD identification. Although biomedical reviews are prospectively registered in PROSPERO, this study was not registered due to the consideration of computational methodology and AI-based COPD identification. The evolving nature of AI research necessitates flexibility in methods and scope, which is not compatible with fixed registration protocols. To mitigate the absence of PROSPERO registration and to preserve methodological credibility, we implemented a predefined governance and amendment plan. The review’s parameters, including objectives, search strategy, eligibility criteria, and primary outcomes, were predetermined and remain constant throughout the process. We identified potential areas for amendments, such as expanding inclusion to preprints and updating search terms to capture emerging AI architectures. Subsequently, we documented the rationale, the exact modification, and its effect on the synthesis. For instance, each adjustment in search vocabulary required a full re-run of the database search and independent dual screening of all new entries. An amendment log was used to document decisions, and the results highlighted the downstream influence of study inclusion and interpretation. Using these strategies, we maintained the core principles of PROSPERO, handling the protocol deviations in a systematic manner to strengthen the trustworthiness of the review process.

### 2.1. Research Questions

This study is designed in order to address the following research questions:

Research question (RQ) 1: What AI techniques and data modalities have been introduced to the identification of COPD?

RQ2: What evaluation strategies and performance metrics are employed to determine the model’s performance?

RQ3: What limitations, biases, and challenges are reported in the existing literature?

### 2.2. Data Sources and Search Strategy

A comprehensive literature search was conducted across multiple biological and technical databases, including PubMed/MEDLINE, Embase, IEEE Xplore, Scopus, and Web of Science. Additionally, arXiv and medRxiv were searched in order to find preprints and upcoming research highlighting the rapid evolution of AI. To capture the increasing importance of deep learning and advanced machine learning in healthcare, this review included studies published between January 2010 and April 2025. Boolean operators, keywords, and controlled vocabulary terms were combined to capture variation in terminology. While the COPD concept was expressed through terms such as “chronic obstructive pulmonary disease,” “COPD,” “chronic bronchitis,” and “emphysema,” AI concepts were captured with terms such as “artificial intelligence,” “machine learning,” “deep learning,” “neural networks,” “support vector machines,” “transformers,” “ensemble methods,” “convolutional neural networks,” and “computer-aided diagnosis.” These were further combined with terms related to diagnostic activity, such as “identification,” “classification,” “screening,” “prediction,” and “detection.”

### 2.3. Study Selection

Table 1 outlines the eligibility criteria for extracting the studies from the databases. The inclusion and exclusion criteria were designed to ensure methodological rigor, clinical relevance, and comparability across the selected studies.

### 2.4. Data Extraction

We used customized syntax according to the database. A reference management system was used to import citations and remove duplicates. Two distinct steps were involved in the selection process. The first step was to evaluate the titles and abstracts in order to exclude irrelevant studies. In the second step, methodological relevance and performance indicators of eligible articles were examined. We retained studies related to AI-based COPD screening that report quantitative performance indicators, including accuracy, sensitivity, specificity, and area under the receiver operating characteristic curve (ROC). Two reviewers conducted independent screening, and disagreements were resolved through consensus.

To ensure consistency across studies, a structured data extraction form was used. Using this form, we extracted variables, including bibliographic information, the country of origin, the data modality (CT, CXR, spirometry, respiratory sounds, and EHR), the size of the dataset, the AI methodology (CNNs, transformers, ensemble methods), preprocessing steps, validation strategies, reported performance metrics, and interpretability approaches (SHAP, Grad-CAM, and feature importance). Additionally, clinical integration, ethical considerations, and data restrictions were recorded.

### 2.5. Risk of Bias and Quality Assessment

We aimed for methodological rigor and openness in assessing the quality of included studies through the application of QUADAS-2 and PROBAST. QUADAS-2 assesses diagnostic accuracy research, whereas PROBAST was utilized to evaluate prediction model studies. The quality of the dataset, the potential for overfitting, the appropriateness of validation procedures, and the precision of outcome definitions were considered during these assessments, enabling a uniform evaluation of methodological rigor across a diverse collection of studies. Reviewers calibrated a subset of studies prior to formal assessment to maintain uniformity in signaling question and applicability domain interpretations. To avoid subjective bias, two reviewers independently evaluated each study using tools and addressed conflicts through consensus discussions throughout the primary review process. In order to formally monitor inter-rater reliability, Cohen’s kappa (κ) was determined for each tool at different stages of the evaluation. The level of agreement across reviewers was confirmed by the substantial agreement (κ > 0.80). After reaching a consensus through structured consensus discussions, any disagreements were acknowledged and documented. We used QUADAS-2 and PROBAST in a systematic and repeatable approach by integrating dual independent assessment, computation of inter-rater agreement metrics, and consensus resolution.

Through systematic evaluation of the included studies, we addressed potential publication biases. By including peer-reviewed literature and preprints from repositories, publication biases were mitigated. The inclusion of preprints supports this study in capturing cutting-edge methodological developments and reducing the risk of time-lag bias. The reported performance indicators were examined for clustering around exceptionally high values, indicating potential issues such as selective reporting, overfitting, or lack of transparency in validation procedures. We reported algorithmic biases, limiting the AI applications in delivering equitable performance across diverse patient populations.

### 2.6. Data Synthesis and Ethical Considerations

We followed a structured narrative synthesis due to the heterogeneity of study designs, AI architectures, dataset sources, and reported outcomes. The included studies were classified based on data modality, reflecting the primary source of information used by AI applications. Under each modality, individual studies were compared on multiple dimensions, including dataset characteristics, methodology, validation strategy, and performance metrics, providing a nuanced understanding of the current landscape of AI-based COPD identification. Moreover, ethical considerations were documented, addressing issues of patient privacy, data protection regulations, and the implications of the studies’ findings for clinical deployment.

Using a PRISMA flow diagram, the study selection processes were documented and visualized, presenting the number of records retrieved from each database, the removal of duplicates, the number of studies excluded during title and abstract screening, the number of studies assessed for full-text eligibility, and the final set of included studies. The explicit reporting of these processes strengthens the reproducibility of this review, synthesizing current evidence and establishing a framework for future studies.

## 3. Results and Discussions

**Figure 1:**
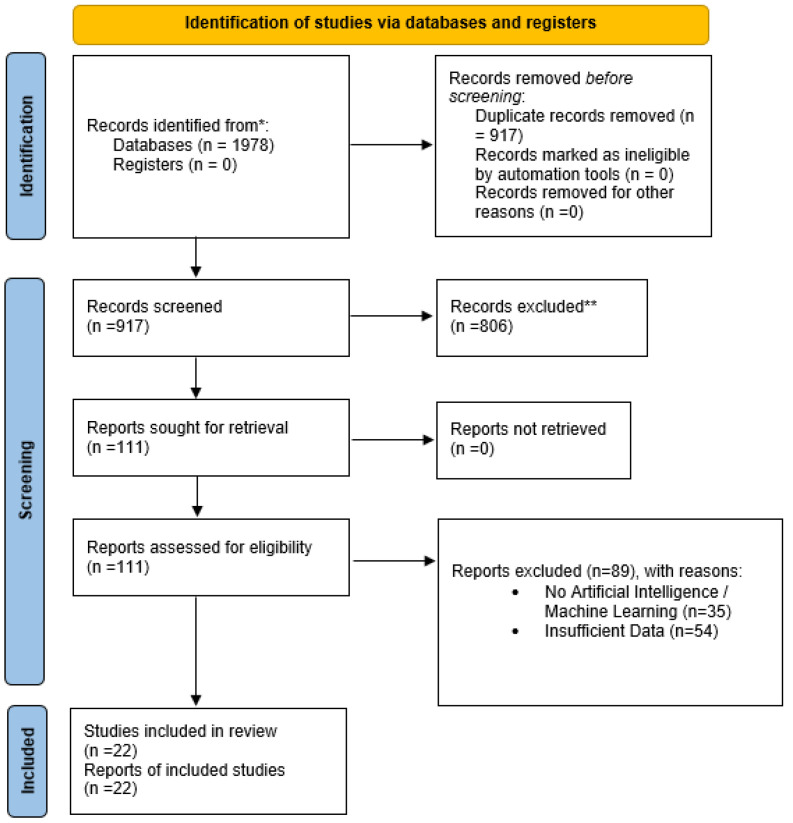
PRISMA-based study selection process. * denotes the primary source and ** denotes the excluded records.

Figure 1 summarizes the review process, ensuring the inclusion of high-quality studies associated with AI-powered COPD identification. A total of 1978 records were identified across multiple databases, reflecting the impact of research studies in medical imaging, machine learning, and computational health sciences. The final set of 22 studies is synthesized in this review, underscoring the rigorous selection process, guaranteeing transparency and reproducibility. The included studies cover various approaches to COPD identification utilizing CT scans, CXR, spirometry, lung sounds, and multimodal data, highlighting the significance of AI in COPD detection. In line with the research objectives, this section is structured around four RQs, outlining the implications, limitations, potential biases, and future research directions of the included studies.

### 3.1. RQ1: What AI Techniques Have Been Introduced to the Identification of COPD?

The synthesis of this review demonstrates the use of diverse data modalities for identifying COPD using distinct methodological innovations based on AI techniques tailored to each modality. The heterogeneity of COPD conditions necessitates adaptive analytical approaches that integrate structural, functional, and symptomatic data. Unlike traditional clinical approaches, AI applications exploit diverse data sources to uncover latent patterns associated with COPD. AI techniques, including conventional machine learning algorithms such as support vector machines (SVMs) and decision trees, as well as more advanced deep learning architectures like convolutional neural networks (CNNs), residual networks, attention-based multiple instance learning (MIL), and self-supervised learning, have been applied to the identification of COPD. RQ1 highlights the diverse range of AI methods for detecting COPD using diverse data modalities. Each modality has its own set of strengths and limitations. CT offers structural accuracy, chest radiography provides scalability, spirometry offers functional insights, and lung sounds/EHR approaches emphasize accessibility and systemic integration. These methods provide the groundwork for evaluating AI’s ability to handle the multidimensionality of COPD.

#### 3.1.1. CT-Based COPD Identification

**Figure 2:**
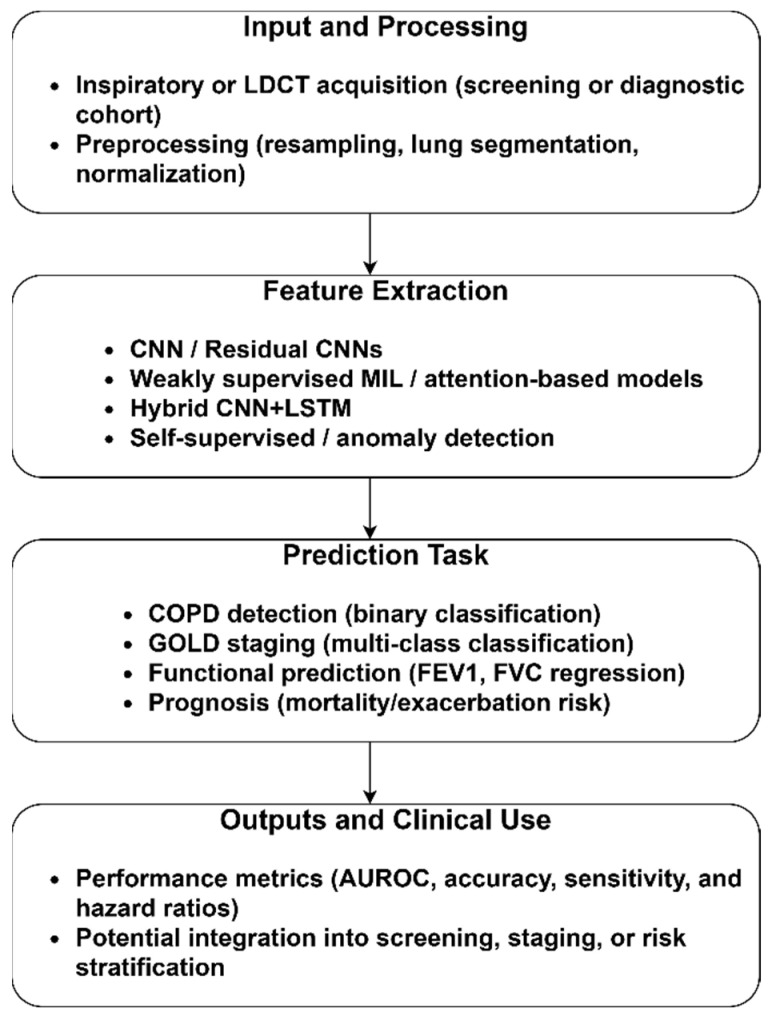
COPD identification using CT images.

The CT modality has been widely used for the identification of COPD due to its capacity to detect emphysema, the thickness of the airway wall, and other structural abnormalities. Figure 2 reveals the sample COPD and normal CT images derived from the Lung Image Database Consortium and Image Database Resource Initiative (LIDC-IDRI) repository [46]. It highlights the extraction of latent structural and functional signatures of COPD from CT images, translating the outcomes into clinically actionable insights. Additionally, it illustrates the structural differences between a normal lung and a lung affected by COPD. The CT features, including emphysema quantification, airway wall thickness, lumen diameter, and measures of air-trapping on inspiratory–expiratory scans, support AI models in differentiating emphysema-predominant and airway-predominant phenotypes.

Figure 2 summarizes the generalized methodological pipeline for COPD identification using low-dose CT (LDCT) scans. Pre-processing steps, including resampling, lung segmentation, and intensity normalization, are crucial for reducing scanner variability and standardizing inputs for downstream algorithms. The feature extraction enables the models to identify subtle COPD patterns. Based on the study’s objectives, the prediction tasks are classified into binary and multi-class classification, regression, and prognosis. Finally, outputs and clinical use are reported using ROC, sensitivity, specificity, accuracy, and hazard ratios. The sample CT images are presented in the Supplementary Material, Figure S1: (a) Normal and (b) COPD CT Images.

Table 2 presents the characteristics of the included studies, including the dataset details, AI approach, validation design, quantitative performance, and limitations. The individual studies highlight the potential of supervised [47,48], weakly supervised [49,50,51,52], and self-supervised [53,54] approaches. The comparative synthesis reveals critical differences in their generalizability. Traditional supervised models [47,48] achieve exceptional outcomes on well-annotated datasets. However, these models show reduced transferability across populations. In contrast, weakly supervised approaches [49,50,51,52] report improved adaptability to partially labeled data, rendering them suitable for real-world deployment. Self-supervised learning strategies [53,54] extend generalizability through robust feature representations that transfer across ethnic and demographic subgroups. Overall, supervised CNN architectures excel in single-center settings, whereas weakly and self-supervised approaches offer resilience to label noise and cross-population diversity, providing a clinically realistic path toward broader implementation.

#### 3.1.2. CXR-Based COPD Identification

The CXR images are widely applied and cost-effective imaging modalities in the evaluation of respiratory diseases. These images are less sensitive compared to the LDCT scans. However, they provide valuable insights into COPD markers. The included studies extract radiographic patterns related to airway obstruction and emphysema. The findings demonstrated that AI-powered CXR analysis can stratify disease severity and predict prognosis, offering a powerful, scalable, and clinically practical approach to early disease detection and risk stratification.

**Figure 3:**
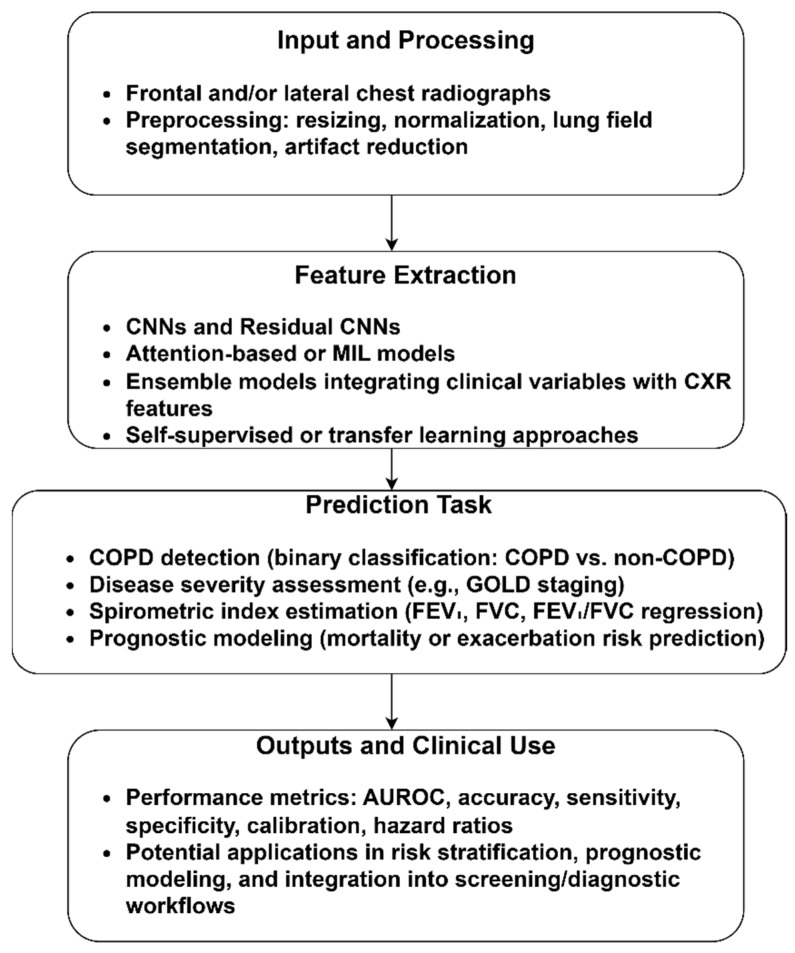
COPD identification using CXR images.

Figure 3 outlines the CXR-based COPD identification pipeline. In the input and preprocessing steps, resizing, normalization, lung segmentation, and artifact reduction were performed to standardize image quality. Feature extraction used diverse deep learning techniques to extract COPD features. Using these features with classification approaches, COPD and non-COPD CXR images were classified. Additionally, disease staging, spirometric index estimation, and prognostic estimates of mortality or exacerbation risk were achieved through the included studies. The model’s performance was evaluated using metrics, including AUROC, accuracy, sensitivity, specificity, calibration, and hazard ratios, rendering the CXR-based COPD identification model a practical tool for large-scale COPD screening and monitoring. The sample CXR images are presented in the Supplementary Material, Figure S2: (a) COPD and (b) Normal CXR Images.

Table 3 highlights the contributions of the included studies based on CXR images, which advance COPD detection and prognosis through the use of AI techniques. The review findings demonstrate the significance of supervised and ensemble learning strategies for CXR-based COPD identification. Early supervised models [55] achieve reliable detection of airflow obstruction. However, the reliance on a single-center dataset introduces institutional bias that restricts its generalizability to diverse healthcare settings. Ueda et al. [56] addressed this limitation by conducting multi-institutional studies. They used regression-based estimation of spirometric indices. However, the variations in image quality may affect the model’s generalization ability. Prognostic models [57,58] expanded the application of CXR-based COPD identification into survival prediction and risk stratification. These models were validated in homogeneous ethnic populations, limiting confidence in broader applicability. The advanced ensemble and multimodal approach [59] outperforms the traditional supervised models through its ensemble learning functionality. These comparisons report the inherent limitations of supervised CNN architectures.

#### 3.1.3. Spirometry/Spirogram-Based COPD Identification

**Figure 4:**
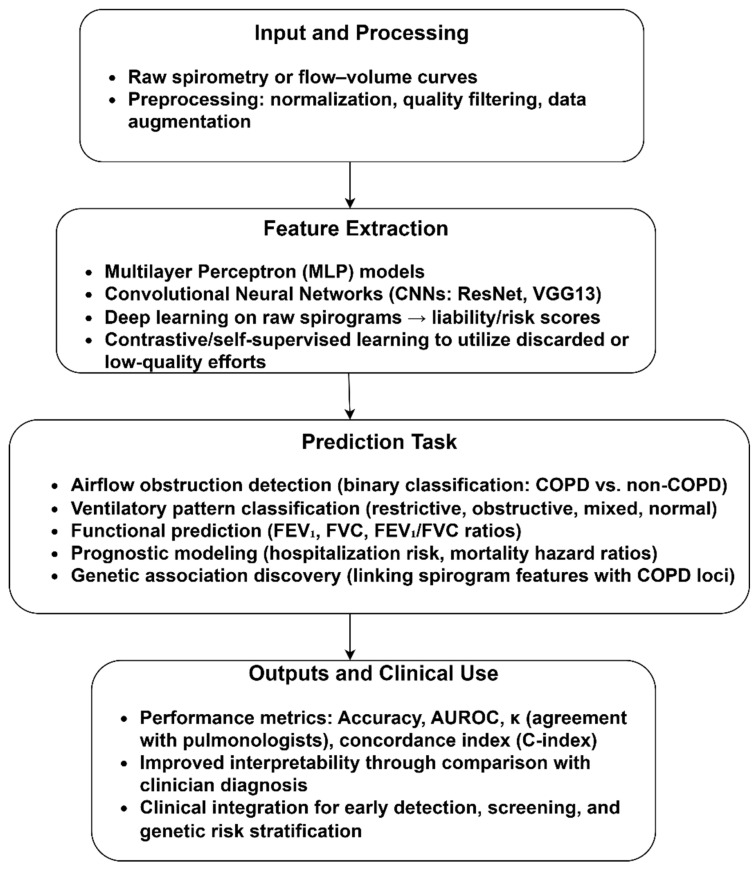
COPD identification using spirogram.

Figure 4 illustrates the significance of spirometry and flow-volume curves in diagnosing and classifying COPD, highlighting the transformation of raw spirograms into clinically meaningful outputs. The preprocessing steps, including normalization, quality filtering, and augmentation, addressed the variability in device calibration. Using MLP, CNNs, and SSL, the included studies enable the exploitation of suboptimal curves. Prediction tasks uncover novel genetic associations, linking respiratory functions to COPD susceptibility loci. Through diagnostic, prognostic, and genetic dimensions, AI-based spirometry holds promise for earlier detection and risk stratification.

Table 4 outlines the significant contributions of the studies based on spirometry and spirogram analysis. Spirometry-based COPD identification approaches highlight the differences between supervised learning and advanced self-supervised strategies. Supervised models [60,61] demonstrate their diagnostic potential by outperforming clinicians in controlled settings. However, the models’ performance relies on data quality and device calibration. Self-supervised and contrastive learning [62,63] address these shortcomings through the extraction of latent features from raw or suboptimal spirograms, improving generalizability across diverse populations. Compared to supervised CNN architectures, self-supervised approaches show exceptional robustness across heterogeneous datasets, rendering them suitable for clinical deployment.

#### 3.1.4. Other Modalities

**Figure 5:**
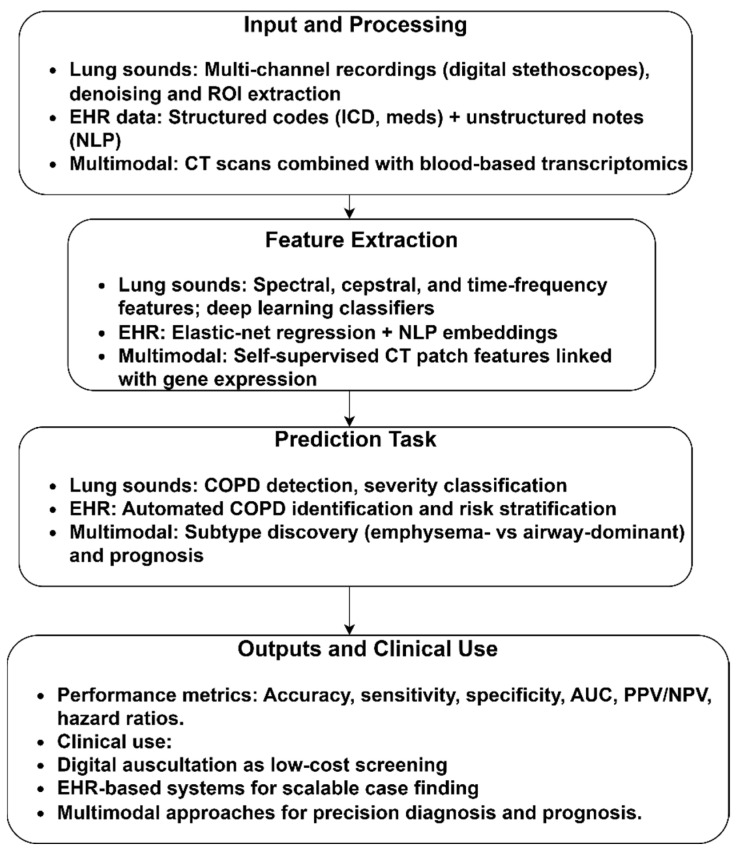
COPD identification using other modalities.

Figure 5 illustrates the importance of lung sounds, EHR, and multimodal imaging in detecting and managing COPD. Using lung sound analysis, features, such as spectral, cepstral, and time–frequency, can be used to identify lung abnormalities, offering a low-cost, non-invasive screening for primary care or resource-limited settings. EHR-based approaches that combine structured data and unstructured clinical notes have demonstrated substantial predictive value for COPD case finding and risk stratification. Unlike an unimodal pipeline, a multimodal model shows the application of CT-derived features with blood transcriptomic profiles, enabling the discovery of clinically relevant subtypes associated with COPD and non-COPD. It highlights underlying biological pathways, advancing precision medicine.

Table 5 summarizes the role of diverse data modalities, including lung sounds, EHR, and multi-omics data, in detecting COPD. The included studies reveal the performance of traditional supervised learning and emerging self-supervised representation learning strategies. Lung sound-based COPD diagnosis and screening strategies [64,65,66] employed supervised models, relying on fused acoustic features. While they report impressive accuracies, the reliance on a single source on public datasets may lead to overfitting and limited generalization. The lack of external validation undermines their translational potential. Additionally, the supervised feature–classifier pipelines tend to learn dataset-specific patterns rather than robust disease signatures. Similarly, Chu et al. [42] demonstrated the performance of a supervised elastic-net logistic regression approach. However, the model’s sensitivity reflects the shortcomings of the supervised model, missing critical COPD features in clinical settings. Chen et al. [67] showed the potential of self-supervised learning through a context-aware representation learning strategy that extracts multimodal image expression with transcriptomic data. Unlike supervised learning, the self-supervised model unveils biologically meaningful subtypes. The cross-validation and independent replication in COPDGene underscore its robustness, uncovering latent disease structure.

### 3.2. RQ2: What Evaluation Strategies and Performance Metrics Are Employed to Determine the Model’s Performance?

The evaluation of AI-driven COPD identification represents a pivotal determinant of its clinical credibility. Although the focus is on algorithmic innovation, the applicability of the included studies relies on the robustness and appropriateness of their evaluations. A wide variety of validation procedures and performance measurements were reported throughout the studies, covering regression and classification. The integration of various methods shows how assessment procedures have progressed while highlighting the gaps that hinder clinical readiness.

The use of internal validation was consistent across modalities. A significant number of studies have employed random train–validation–test splits or k-fold cross-validation in order to quantify the model’s performance on unseen data. This method was widely used in CXR and spirometry-based studies due to the relatively smaller datasets and the need to make optimal use of the data. In the attention-based CT model, Xue et al. [50] applied cross-validation to minimize the sampling variance and optimize the amount of knowledge gained from a small number of cohorts. Tang et al. [47] validated the pre-trained model, reporting stable AUROC values of ~0.89. Similarly, Schroeder et al. [57] and Zou et al. [59] applied the AUROC metric to evaluate the chest radiography-based COPD detection approaches. Sun et al. [49] and Almeida et al. [53,54] employed multicenter and multi-ethnic cohorts, highlighting the importance of evaluation strategies incorporating fairness and inclusivity. Park et al. [51] reported the mean absolute error and concordance correlation coefficients in order to evaluate CT-based prediction. Ueda et al. [56] extended this framework by including intra-class correlation coefficients and Pearson correlations. Gonzalez predicted mortality using hazard ratios.

Due to their dual role in diagnosis and prognosis, CT studies typically emphasized AUROC and hazard ratios. CXR studies employed regression measures to estimate spirometric indices. Spirometry-based models prioritize sensitivity and specificity. Due to the limited size of the datasets, lung-sound models have maintained high levels of accuracy and AUROC. However, these studies lack external validation. In order to highlight their incorporation of structured and unstructured clinical data, EHR-based and multimodal models focused on specificity, PPV, and incremental AUROC. A significant improvement over relying on AUROC is the incorporation of prognostic indicators, multicenter diversity, and external validation. The majority of the included studies were in the preparation stage rather than being ready for clinical deployment due to the inconsistent use of calibration, fairness testing, and clinical utility metrics. Limited validation in homogeneous groups increases the likelihood that models may underperform in diverse clinical contexts. High AUROC scores without calibration or decision-curve analysis may lead to inflated confidence and obscure practical limitations. Overall, the findings highlight the lack of standardized assessment frameworks.

### 3.3. RQ3: What Limitations, Biases, and Challenges Are Reported in the Existing Literature?

The AI-based COPD literature exhibits methodological and practical limitations, including dataset and spectrum bias, acquisition and label heterogeneity, retrospective design, class imbalance, and limited fairness and interpretability.

CT-based COPD screening relies on cohorts enriched with smokers, such as COPDGene, ECLIPSE, or lung cancer screening programs. These datasets strengthen the detection of emphysema and airway abnormalities, restricting the models’ applicability to non-smokers and underdiagnosed populations. CXR studies were based on specific countries or health systems, leading to a spectrum bias that limits their generalizability. The reliance on the UK Biobank dataset limits the applicability of the spirogram-based models. The lung sound datasets were small and device-specific. Similarly, EHR-driven approaches depend on the specific coding and documentation practices, presenting challenges in deploying these models in heterogeneous settings.

The heterogeneity in acquisition and labeling has the potential to generate domain shift and complicates benchmarking. A wide variety of scanner manufacturers and reconstruction kernels are considered in CT databases, which contain the inspiratory and expiratory phases. In the absence of harmonization, models can mistakenly learn scanner artifacts rather than disease signatures. The reliability of the CXR-based models may be compromised by differences in projection, equipment (portable vs. stationary), and patient position. In contrast to lung-sound recordings, which vary in terms of microphone quality, channel number, and background noise, spirometry is highly dependent on the patient’s efforts and the calibration of the specific equipment. EHR-based studies face challenges, including uneven categorization, various note styles, and missing information. In addition, there is an inconsistency in reference labeling. The fixed-ratio criteria exaggerate the prevalence in older persons, the GOLD staging suffers from moderate repeatability, and the code-based phenotyping approach causes misclassification. Spirometry is not foolproof due to time discrepancies between image or sound capture and lung function measurement, reducing comparability across studies.

Study design limitations undermine the trustworthiness of the studies’ findings. The majority of the studies were based on a retrospective dataset. A few CT and CXR models used independent external cohorts, while many utilized internal splits with cross-validation. There was a lack of prospective assessment across the modalities. Dependence on retrospective processes leads to data leakage, especially when multiple images from the same patient are included in both training and test sets, or when hyperparameters are accidentally tuned on test data. Another widespread challenge is class imbalance. Most studies reported overall accuracy or AUROC, leading to poor sensitivity or specificity in minority subgroups. There was a lack of reporting of precision–recall measures, F1-scores, calibration plots, and decision-curve studies, leading to misinterpretation of the model’s performance. The incorporation of saliency maps or attention overlays into some CT and CXR models has been identified. However, these representations tend to be unstable and have not been thoroughly evaluated against radiological markers. In the spirogram and lung-sound-based studies, the physiological or acoustic correlates of model predictions were rarely clarified. As a result, clinicians may face challenges in making informed decisions based on the findings of these models. EHR approaches rely on documentation artifacts rather than disease biology, reducing their interpretability and explainability.

Across the studies, another critical gap is the limited use of robust external validation. For instance, CT and CXR-based models were trained and validated on hospital-specific datasets. These models may encounter challenges in handling diversity in scanner protocols, demographic distributions, and disease prevalence. Models trained on COPDGene and ECLIPSE datasets demonstrate improved robustness across populations and acquisition settings. The UK Biobank dataset facilitates the training and discovery of genetic associations using spirometry-based models for COPD identification. However, the ethnic homogeneity restricts the model’s applicability to non-European populations. EHR-based models-based on two U.S. healthcare datasets, indicate the importance of cross-institutional validation. However, the differences in practice-specific coding may impact the model’s performance. These findings underscore the need for large, multi-institutional datasets with demographic and geographic diversity.

The included studies lack a comprehensive assessment of the subgroup. Despite reporting aggregate accuracy or AUROC, limited studies have assessed performance across age, sex, ethnicity, or geography, which are crucial to generalizability and fairness. As a result of this exclusion, there is a possibility of overlooking systemic biases that might impair equitable clinical deployment. For instance, the spirometry model using the UK Biobank dataset [61,63] benefitted from a large sample size and high-quality measurement. However, the data were derived from a predominantly White British cohort. Ethnic homogeneity poses uncertainties regarding applicability to non-Europeans with different lung function norms, smoking behaviors, and COPD prevalence. In the absence of rigorous subgroup validation, these models have the potential to incorrectly categorize underrepresented groups. The majority of CT and CXR models were trained on older male smokers, restricting their applicability to women, younger individuals, and never-smokers with distinct disease trajectories. Due to datasets derived from single institutions or local health systems, lung-sound and EHR-based techniques confront similar challenges. Variations in clinical practice, diagnostic labeling, and environmental exposures may result in the inability of predictors learnt in these settings to transfer consistently across other geographic or cultural contexts.

The significance of subgroup analysis is shown by concrete examples from other domains of AI-based medical diagnosis. The use of comparable subgroup reporting in COPD research would reveal hidden biases and facilitate the implementation of remedial procedures. Methodological improvements are needed to address subgroup performance. The strategies, including targeted data augmentation for underrepresented groups, fairness-aware loss functions during training, or transfer learning, can be used to improve COPD-identification models’ performance across regions and cohorts. Interpretability tools, such as SHAP values and attention overlays, can reveal subgroup-specific feature dependencies, such as the distribution of emphysema across ethnic groups.

### 3.4. Future Directions

AI-driven COPD diagnosis and categorization have demonstrated significant potential across imaging, physiological, acoustic, and EHR modalities. Future research should adhere to a staged roadmap that prioritizes short-term feasibility, mid-term consolidation, and long-term innovation in order to transform the methodological advancements into therapeutically beneficial and clinically trusted tools.

In the short term, reducing spectrum bias and strengthening external validation are the key priorities. Assessing AI models in a real-world environment requires prospective, multicenter studies. To promote fairness and worldwide applicability, these initiatives should target underrepresented populations, including women, younger individuals, never-smokers, and low- and middle-income patients. Additionally, it is essential to implement standardized reporting frameworks, such as TRIPOD-AI, PROBAST-AI, and CONSORT-AI, to foster transparency, comparability, and reproducibility.

The mid-term research should emphasize multimodal integration and interpretability. We identified a potential combination of modalities for developing multimodality-based COPD diagnosis and screening. The complementary value of structural and functional information is shown by CXR-derived features combined with spirometry or clinical data, outperforming imaging-only models in staging and prognostic tasks. The integration of genomic or transcriptome data with CT-based models has shown promise for extending precision medicine beyond diagnosis by revealing molecular pathways and identifying subtypes of COPD. Combining EHR-driven models with spirometry can improve prediction accuracy, addressing coding variability. Although the lung-sound classifiers deliver reasonable performance with limited computational costs, they remain underexplored in multimodal contexts. By combining structural and functional modalities and clinical phenotypes with molecular profiles, an effective COPD identification model can be developed. Future research should evaluate integrative approaches in multicenter, demographically diverse cohorts and explicitly correlate AI-derived features with pathophysiological markers, such as airway wall thickening, emphysema subtypes, and acoustic wheeze signatures, to enhance the interpretability of COPD identification models.

Long-term research should focus on addressing fairness, sustainability, and clinical application. Sustainable computing approaches and energy-efficient architectures should be standard procedures in future work to mitigate the environmental impacts of training AI models on a broader scale. In order to guarantee an equitable and clinically reliable deployment, it is recommended that future research on COPD artificial intelligence include comprehensive subgroup studies (based on age, gender, ethnicity, and geographic location) and combine them with interpretability frameworks. AI-derived outputs, such as risk score, PFT values, or imaging-derived severity indices, can support current diagnostic workflows through quantitative decision support. Using this integration, personalized treatment planning and earlier diagnosis can be streamlined. However, the barriers, including a lengthy regulatory approval process, ethical challenges, and limited infrastructure, should be addressed in order to facilitate the seamless adoption. With extensive external validation, multimodal, interpretable models, and fairness-aware, sustainable healthcare systems, promising research may be transformed into reliable, equitable, and globally deployable AI solutions for COPD diagnosis and treatment.

### 3.5. The Review’s Limitations and Potential Biases

The limitations of this study should be addressed to contextualize its findings. Publication bias may exist despite a comprehensive multi-database search strategy. High-performance measures or unique approaches are likely to be published, whereas negative or underperforming data may be unpublished, leading to overestimation of AI model efficacy. The review was limited to English-language publications that may introduce language bias. This may have excluded potentially relevant studies from other languages, primarily from regions with high COPD prevalence. AI architectures, data acquisition protocols, labeling standards, and outcome measures varied across the included studies, limiting the feasibility of a quantitative meta-analysis. While narrative synthesis provides a qualitative overview, it reduces the comparability of the performance of the included studies. The inclusion of preprints ensured coverage of the recent developments in COPD screening and diagnosis. However, these studies typically lack the rigor of peer review. These studies may change their findings upon further analysis or methodological refinements, introducing uncertainty to the evidence base. Furthermore, the possibility of time-lag bias exists in any review that delves into an emerging field of study, presenting a current snapshot rather than a final and comprehensive account of AI applications in COPD detection due to the exclusion of recently published or ongoing studies.

## 4. Conclusions

This review synthesized 22 studies covering CT, CXR, spirometry, lung sounds, and EHR-based approaches, enabling the detection and prognosis of COPD. CT-based studies demonstrated discriminative ability, underscoring their prognostic potential. CXR-based studies have shown promising outcomes, supporting population-level screening and providing a reliable estimate of spirometric indices. Spirometry-driven approaches achieved exceptional accuracy in detecting airflow obstruction. Emerging modalities, including lung sounds, EHR, and multimodal data, illustrated the capability of AI in harnessing non-imaging data to identify COPD-associated risks. However, the majority of these studies were retrospective, which reduced their representativeness and global applicability. Methodological limitations, such as inconsistent external validation and insufficient subgroup analysis to assess fairness across the population, hinder clinical trust and adoption. This review acknowledges its limitations. The dependence on the English-language studies may have excluded relevant non-English studies. The heterogeneity in methodological approaches and performance metrics prevents a formal meta-analysis. The findings of this review set a pathway for future research. Prospective validation and multicenter datasets are required to guarantee fairness and generalizability. Incorporating multimodal data can capture the multifaceted dimensions of COPD, improving risk prediction.

## Figures and Tables

**Table 1:** Inclusion and exclusion criteria.

Inclusion	Exclusion
Original research articles and preprints with methodological detail.	Narrative reviews, systematic reviews, and conference abstracts that lack sufficient data.
Studies based on human-derived datasets.	Animal studies and simulated data without clinical relevance.
AI-based COPD detection, classification, or diagnosis.	Statistical models without algorithmic learning.
Studies reporting quantitative performance metrics.	Studies without measurable performance outcomes.
Studies published in the English language between 2010 and 2025.	Non-English publications.

**Table 2:** Findings of CT-based COPD identification approaches.

Study	Dataset Number of Participants (*n*)	AI Approach	Validation Design	Quantitative Performance	Limitations
Tang et al. [47]	Training Dataset: *n* = 2589 (Male: 1440 and Female: *n* = 1149) Location: Canada External Dataset: *n* = 2195 (Male: 1318 and Female: 877) Location: ECLIPSE (Multinational)	Fine-tuned Residual CNNs architecture	Three-fold cross-validation (CV) on PanCan; external test on ECLIPSE with no model change.	CV-AUC 0.889 ± 0.017; external AUC 0.886 ± 0.017; positive predictive value (PPV) 0.847 ± 0.056, negative predictive value (NPV) 0.755 ± 0.097	Screening bias, retrospective, and limited generalization
González et al. [48]	*n* = 7983 COPDGene (US, multicenter smokers) + *n* = 1672 ECLIPSE (multinational smokers)	2D CNN using four canonical CT views for COPD detection, Global Initiative for Chronic Obstructive Lung Disease (GOLD) staging, and prognosis.	Internal test in COPDGene; external test in ECLIPSE; logistic/Cox models for events & mortality.	AUC = ~0.85 for COPD Accuracy = 74.95%	Smoker-only cohorts and cross-study CT protocol differences.
Sun et al. [49]	Multicenter China CT cohort: *n* = 1393 from four large public hospitals; labels from spirometry; external validation on NLST subset (~620 cases).	Weakly supervised CT DL (multiple-instance/patch-bag paradigm) for COPD detection + GOLD staging.	Internal split in the Chinese cohort; external check on National Lung Screening Trial (NLST).	GOLD grading accuracy ≈ 76.4% with weighted κ ≈ 0.619	Retrospective and site-specific protocols.
Xue et al. [50]	*n* = 800 for training and testing. *n* = 260 for the external validation	Two-Stage-Attention MIL (TSA-MIL) using slice/patch bags; compares against conventional MIL and CNN baselines.	Cross-validation; external test set included.	Internal accuracy ≈ 92%, AUC ≈ 0.95; external AUC ≈ 0.87	Potential label noise from weak supervision, single-country data, and limited reporting on scanner harmonization.
Park et al. [51]	*n* = 16,148 with mean age of 55 ± 10 (standard deviation)	Volumetric CT deep learning and regression/classification techniques to predict PFTs (FEV_1_, FVC, FEV_1_/FVC) and flag abnormality.	Train/validation/test splits, internal validation, and threshold-based screening analyses.	Reported sensitivities were 61.6%, 46.9%, and 36.1% for different abnormality thresholds.	Single-country screening population and limited generalization.
Humphries et al. [52]	COPDGene test cohort *n* = 7143; external ECLIPSE *n* = 1962; US/multinational smokers with baseline inspiratory CT and outcomes.	CNNs + LSTM to automate emphysema grading on CT.	Internal testing (COPDGene) + external testing (ECLIPSE).	Weighted κ (DL vs. visual) 0.60; mortality hazard ratios vs. “no emphysema”: 1.5, 1.7, 2.9, 5.3, 9.7	Smoker-enriched cohorts and demand substantial computational resources.
Almeida et al. [53]	COPDGene: train/val/test *n* = 3144/*n* = 786/*n* = 1310; external COSYCONET *n* = 446 (Germany).	SSL representation + anomaly-detection on 3D CT patches to quantify COPD severity as “anomaly score.”	Internal test (COPDGene) and external test (COSYCONET); compared to supervised DL baselines; linked anomaly score to PFTs, SGRQ, emphysema, and air-trapping.	AUC = 84.3 ± 0.3 (COPDGene) and AUC = 76.3 ± 0.6 (COSYCONET) for COPD versus low-risk; the anomaly score is significantly associated with lung function, symptoms, and exacerbations (*p* < 0.001).	Scanner/protocol variability and anomaly approach improve generalization. However, interpretability and thresholds for clinical use are in an emerging stage.
Almeida et al. [54]	COPDGene: train/val/test *n* = 3144/*n* = 786/*n* = 1310	SSL versus Supervised CNN for COPD Detection: Uncertainty Estimation and Fairness Analysis.	Cross-ethnic internal/external style evaluation.	SSL showed significantly higher AUC than supervised across ethnic groups (*p* < 0.001; article emphasizes generalization rather than a single pooled AUC).	US-centric cohorts, limited emphasis on generalization and fairness metrics.

**Table 3:** Findings of CXR-based COPD identification approaches.

Study	Dataset Number of Participants (*n*)	AI Approach	Validation Design	Quantitative Performance	Limitations
Nam et al. [55]	4225 patients Training (*n* = 3475), validation (*n* = 435), and test (*n* = 315)	DL survival model (DLSP_CXR and integrated DLSP_integ). Outcomes via time-to-event modeling.	Development + validation cohorts; time-dependent AUC analyses vs. established indices.	The time-dependent AUC of the DL model showed no difference compared to BODE (0.87 vs. 0.80; *p* = 0.34), ADO (0.86 vs. 0.89; *p* = 0.51), SGRQ (0.86 vs. 0.70; *p* = 0.09), and was higher than CAT (0.93 vs. 0.55; *p* < 0.001); good calibration.	Prognostic (not diagnostic), Korean cohorts, and treatment decisions require prospective testing.
Ueda et al. [56]	Multi-institution Japan; 81,902 patients with 141,734 CXR-spirometry pairs from five hospitals and external tests at two independent institutions (*n* = 2137 CXRs; *n* = 5290).	DL model to estimate FVC and FEV1 from a single CXR (regression).	Training/validation/testing on three sites, external testing on two sites.	FVC r = 0.91/0.90, ICC = 0.91/0.89, MAE = 0.31 L/0.31 L; FEV1 r = 0.91/0.91, ICC = 0.90/0.90, MAE = 0.28 L/0.25 L.	Retrospective, Japan-only cohorts, and estimate lung function (not a COPD label).
Schroeder et al. [57]	Single-institution (USA); 6749 two-view CXRs (2012–2017) from 4436 subjects with near-concurrent PFTs (≤180 days).	ResNet-18 (frontal + lateral) trained on PFT labels to predict airflow obstruction. Compared with an NLP model of radiology reports.	Training/validation/testing subject splits.	AUC 0.814 for obstructive lung disease (FEV1/FVC < 0.70); for severe COPD (FEV1 < 0.5), AUC 0.837; both > NLP (0.704 and 0.770; *p* < 0.001).	Single-center, retrospective, and limited generalizability.
Doroodgar Jorshery et al. [58]	Ever-smokers (*n* = 12,550) (mean age 62·4 ± 6·8 years, 48.9% male, 12.4% rate of 6-year COPD) and never-smokers (*n* = 15,298) (mean age 63.0 ± 8.1 years, 42.8% male, 3.8% rate of 6-year COPD), collected at Massachusetts General Brigham (MGB) hospital, Boston, USA.	Uses pre-trained CXR-Lung-Risk CNN to stratify risk for incident COPD from a single baseline CXR.	External retrospective validation; time-to-event analyses for incident COPD.	Reports risk stratification for 6-year incident COPD; (focuses on HRs/C-indices rather than diagnostic AUC)	Retrospective design and single-site primary cohort.
Zou et al. [59]	Multicenter China; 1055 participants (COPD *n* = 535, controls *n* = 520) with frontal CXR + clinical data; internal test *n* = 284; external test *n* = 105.	Ensemble DL combining CXR features + clinical parameters for COPD screening and GOLD staging.	Internal split + external test from another site.	COPD detection AUC: internal 0.969 (fusion), external 0.934; CXR-only 0.946; clinical-only 0.963. Staging AUC: 0.894 (3-class) and 0.852 (5-class).	Retrospective; modest external cohort; exclusion of many comorbid lung diseases may overstate real-world performance.

**Table 4:** Findings of Spirometry-based COPD identification approaches.

Study	Dataset Number of Participants (*n*)	AI Approach	Validation Design	Quantitative Performance	Limitations
Mac et al. [60]	~1400 patients, Toronto General Hospital, Canada; single-center cohort with spirometry + plethysmography	Multilayer perceptron (MLP) deep learning vs. pulmonologists	Internal validation with cross-validation; performance compared against clinicians and PFT “gold standard”	Outperformed pulmonologists in classification; comparable to complete PFT diagnostic acumen (exact accuracy not specified, κ > 0.7 reported)	Single-center, relatively small sample, limited external validation, restricted to Canadian patients, and may not capture device/institutional variability
Wang et al. [61]	18,909 subjects, First Affiliated Hospital of Guangzhou Medical University, China; large hospital-based PFT dataset	CNN-based models (ResNet, VGG13) on flow–volume curves	Split into training, validation, and test sets; compared DL vs. pulmonologists and primary care physicians	VGG13 accuracy 95.6%; pulmonologists 76.9% (κ = 0.46); primary care physicians 56.2%	Single-institution dataset, Chinese-only population, retrospective, limited interpretability, imbalance across ventilatory patterns
Cosentino et al. [62]	~350,000 participants, UK Biobank, United Kingdom; raw spirograms (ages 40–69, general population)	Deep learning on raw spirograms → COPD liability score	Training/validation/test splits within UK Biobank; evaluated against traditional phenotypes and genetic associations.	AUROC ~0.82 for COPD; AUROC ~0.89 for COPD hospitalization; HR ~1.22 for mortality prediction; identified 67 novel loci.	Labels are noisy due to their reliance on EHR/ICD codes, a UK-only cohort (limited generalizability), no bronchodilator data, and limited ethnic diversity.
Hill et al. [63]	~350,000 participants, UK Biobank, United Kingdom; included QC-passed and suboptimal spirograms.	Contrastive learning (Spiro-CLF) using all efforts, incl. discarded curves.	Train-test split within the UK Biobank and held-out test set.	AUROC of 0.981 for obstruction; C-index 0.654 for mortality (vs. ~0.59 best effort only).	UK-only cohort (limited generalizability), limited ethnic diversity, and retrospective.

**Table 5:** Findings of other modalties-based COPD identification approaches.

Study	Dataset Number of Participants (*n*)	AI Approach	Validation Design	Quantitative Performance	Limitations
Altan et al. [64]	41 patients underwent 12-channel auscultations (posterior/anterior chest points) in Turkey.	Feature engineering through 3D second-order difference plots (chaos plots); quantization; Deep Extreme Learning Machine.	Retrospective single-dataset development; internal evaluation only (no independent external site).	Overall accuracy 94.31%, weighted sensitivity 94.28%, weighted specificity 98.76%, AUC 0.9659 for five-class COPD severity.	Small, single-source dataset, no external validation, and explicit external testing not reported.
Naqvi & Choudhry [65]	The International Conference on Biomedical and Health Informatics (ICBHI) dataset comprises 920 recordings and 126 subjects in total. The study subset utilized 703 recordings for the COPD, pneumonia/healthy classes, making it a multi-site public dataset.	Region of Interest (ROI) extraction + denoising, fusion of time/cepstral/spectral features; backward elimination feature selection; classifiers compared.	Internal cross-validation and hold-out evaluation on ICBHI; no self-collected or clinical external cohort	Best model: Accuracy 99.70%, TPR > 99%, FNR < 1% on selected fused features	Limited patient metadata, public mixed-source recordings, potential label/device noise, and no external clinical validation.
Yu et al. [66]	12-channel lung-sound recordings from 42 COPD patients (age 38–68; 34 men/8 women). Data recorded by pulmonologists with Littmann 3200.	Hand-engineered Hilbert–Huang Transform (EEMD) time–frequency–energy features → ReliefF channel/feature selection → SVM classifier (compared to Bayes, decision tree, DBN). Uses 4 channels (L1–L4) after selection.	Retrospective model development on the public dataset; experiments compare multi-class tasks (mild vs. moderate + severe; moderate vs. severe). Internal resampling; no external cohort.	For mild vs. moderate + severe: Accuracy 89.13%, Sensitivity 87.72%, Specificity 91.01%. For moderate vs. severe: Accuracy 94.26%, Sensitivity 97.32%, Specificity 89.93%.	Small sample, single public dataset, and no external validation.
Chu et al. [42]	Mass General Brigham (MGB) Biobank (Boston, MA)—3420 screen-positive candidates, gold-standard chart-review labels by pulmonologists; train/test gold standards *n* = 182/100 (77/46 COPD cases). External validation: Marshfield Clinic (Wisconsin) independent EHR/biobank sample.	Elastic-net logistic models using structured EHR (ICD, meds, etc.) + NLP features (SAFE) from notes; compared models with/without spirometry features.	Internal testing on MGB gold-standard test set; independent external validation at Marshfield with identical screening/filtering and blinded chart review.	Internal MGB: PPV 91.7%, Sensitivity 71.7%, Specificity 94.4%. External (Marshfield): PPV 93.5%, Sensitivity 61.4%, Specificity 90%.	Trained in biobank/EHR populations; sensitivity drops without spirometry; portability shown across two U.S. systems; However, international generalizability is not investigated.
Chen et al. [67]	1223 participants with inspiratory/expiratory CT + blood RNA-seq (50% female; 82% NHW, 18% African American; mean age 67). Train/test split 923/300; additional independent replication on 1527 COPDGene participants with CT but without RNA-seq. The Siemens B31F kernel is included for analysis.	CT patch features via context-aware self-supervised representation learning (CSRL) → MLP to learn image-expression axes (IEAs) linking CT structure and blood transcriptome; associations tested with clinical traits and Cox survival models; pathway enrichment.	Nested 5-fold CV within training; held-out test set; independent replication in non-RNA-seq COPDGene subset; focuses on associations and prognosis rather than binary classification.	Identified emphysema-predominant, airway-predominant strongly correlated with CT emphysema/airway measures and lung function; significant associations with outcomes via Cox models and enriched biological pathways (adjusted *p* < 0.001).	Single vendor/kernel (Siemens b31f) constraint; U.S. smoker cohort; not a straightforward “COPD vs. non-COPD” classifier; generalizability beyond COPDGene not demonstrated.

## Data Availability

Not applicable.

## Supplementary Materials

The following supporting information can be downloaded at: https://www.mdpi.com/article/10.3390/diagnostics15202562/s1, Figure S1: (a) Normal and (b) COPD CT Images; Figure S2: (a) COPD and (b) Normal CXR Images.
