# Supplementary figures and images for: Artificial Intelligence-Powered Chronic Obstructive Pulmonary Disease Detection Techniques—A Review
Source: Diagnostics (Basel). 2025 Oct 11;15(20):2562. doi: 10.3390/diagnostics15202562 (PMC12563702; doi:10.3390/diagnostics15202562)

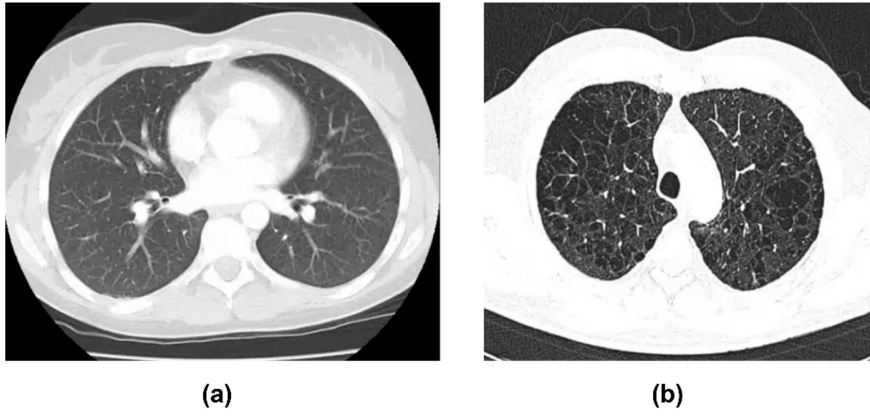

**Figure S1.** (a) Normal and (b) COPD CT Images.

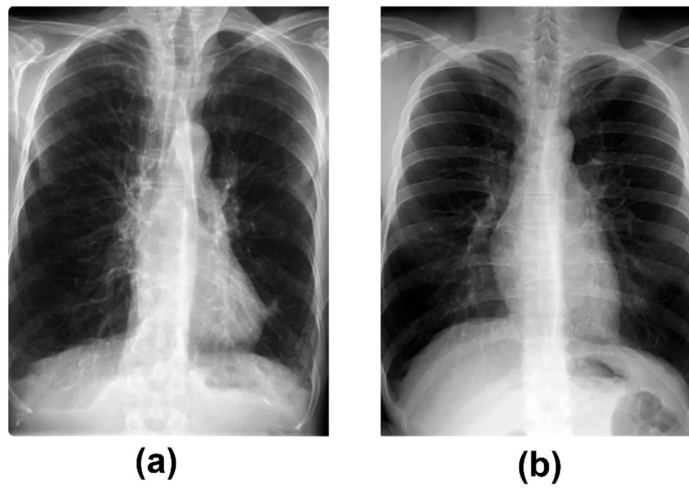

**Figure S2.** (a) COPD and (b) Normal CXR Images.

Supplement: Supplementary file 1 [file diagnostics-15-02562-s001.zip › diagnostics-3907849-supplementary.pdf]
